# CD8^+^ T cell gene expression analysis identifies differentially expressed genes between multiple sclerosis patients and healthy controls

**DOI:** 10.1177/2055217320978511

**Published:** 2020-12-09

**Authors:** IS Brorson, AM Eriksson, IS Leikfoss, V Vitelli, EG Celius, T Lüders, T Berge, HF Harbo, H Nilsen, SD Bos

**Affiliations:** Institute of Clinical Medicine, University of Oslo, Oslo, Norway; Department of Neurology, Oslo University Hospital Ullevål, Oslo, Norway; Oslo Centre for Biostatistics and Epidemiology, Department of Biostatistics, University of Oslo, Oslo, Norway; Institute of Clinical Medicine, University of Oslo, Oslo, Norway; Department of Neurology, Oslo University Hospital Ullevål, Oslo, Norway; Department of Clinical Molecular Biology, University of Oslo and Akershus University Hospital, Oslo, Norway; Department of Mechanical, Electronics and Chemical Engineering, Oslo Metropolitan University, Oslo, Norway; Department of Research, Innovation and Education, Oslo University Hospital, Oslo, Norway; Institute of Clinical Medicine, University of Oslo, Oslo, Norway; Department of Neurology, Oslo University Hospital Ullevål, Oslo, Norway; Department of Clinical Molecular Biology, University of Oslo and Akershus University Hospital, Oslo, Norway; Institute of Clinical Medicine, University of Oslo, Oslo, Norway; Department of Neurology, Oslo University Hospital Ullevål, Oslo, Norway

**Keywords:** Multiple sclerosis, T-Lymphocytes, gene expression, RNA, autoimmunity, neurology

## Abstract

**Background:**

Genetic and clinical observations have indicated T cells are involved in MS pathology. There is little insight in how T cells are involved and whether or not these can be used as markers for MS.

**Objectives:**

Analysis of the gene expression profiles of circulating CD8^+^ T cells of MS patients compared to healthy controls.

**Methods:**

RNA from purified CD8^+^ T cells was sequenced and analyzed for differential gene expression. Pathway analyses of genes at several p-value cutoffs were performed to identify putative pathways involved.

**Results:**

We identified 36 genes with significant differential gene expression in MS patients. Four genes reached at least 2-fold differences in expression. The majority of differentially expressed genes was higher expressed in MS patients. Genes associated to MS in GWAS showed enrichment amongst the differentially expressed genes. We did not identify enrichment of specific pathways amongst the differentially expressed genes in MS patients.

**Conclusions:**

CD8^+^ T cells of MS patients show differential gene expression, with predominantly higher activity of genes in MS patients. We do not identify specific biological pathways in our study. More detailed analysis of CD8^+^ T cells and subtypes of these may increase understanding of how T cells are involved in MS.

## Introduction

Multiple sclerosis (MS) is characterized by immune cell infiltration and inflammation of the central nervous system (CNS) leading to loss of neurological function. Recent advances in genetics also indicate a large role of the immune system in the genetic risk factors,^1^ in addition to epigenetic^2,3^ and environmental factors.^4^ T-helper cell specific pathways have been shown to be involved through pathway analysis of the genetic risk variants.^5^ Further corroboration for T cell involvement is provided by biological pathway and gene-set enrichment analyses of MS genetic and genomic studies.^6^ Whilst CD4^+^ T cells have been considered to be major contributors in the pathological processes observed in MS,^7–10^ histological examinations of MS lesions show that CD8^+^ T cells outnumber CD4^+^ T cells in active lesions.^11,12^ Further studies on the role of CD8^+^ T cells in MS indicate that these cells may act as disease effectors.^13,14^

Earlier studies of gene expression in MS performed in whole blood and peripheral blood mononuclear cells (PBMCs) have not shown large scale differences between MS patients and healthy controls.^15–17^ A study of whole blood gene expression by Parnell *et al.*, comparing MS patients to healthy controls showed differential gene expression of *EOMES* and *TBX21.*^17^ Despite clear evidence for involvement of T cells illustrated by the effectiveness of treatments that target T cells, we did not detect gene expression differences for CD4^+^ T cells of untreated MS patients compared to healthy controls.^18^

Whilst genome-wide association studies (GWAS) are not affected by a putative patient treatment, the gene expression profiles are affected by immunosuppressive and immunomodulatory drugs in use for treatment of MS. In our study, we therefore included untreated MS patients only. We performed RNA sequencing of peripheral CD8^+^ T cells from 20 MS patients and 20 healthy controls to identify genes that are differentially expressed by these cells in MS patients.

## Materials and methods

### Patients and healthy controls

Female MS patients with relapsing remitting MS and no other autoimmune disorders (N = 20) were recruited from the MS clinic at the Oslo University Hospital. 18 of these patients never received immunomodulatory treatment for MS, whereas two patients underwent interferon-beta treatment that was stopped respectively two and four years prior to inclusion in this study (Supplementary Table S2). None of the patients was treated with any medication at the time of inclusion in this study. All patients met the 2010 McDonald diagnostic criteria.^19^ Unrelated female, age-matched (N = 20) healthy controls were recruited from the patients’ social network or hospital employees. All patients and healthy controls provided informed consent for this study, which was approved by the local medical ethical committee (REK2011/1846).

### Purification of CD8^+^ T cells

Whole blood was drawn into EDTA coated vacuum tubes (Med-Kjemi AS, Norway). PBMCs were purified within two hours of venipuncture using lymphoprep (Axis Shield, Scotland). The PBMCs were washed with PBS and resuspended at a density of 1*10^8^ cells per ml in purification buffer (1 mM EDTA and 2% FCS in PBS).

CD8^+^ T cells were isolated by positive selection using an Automacs cell separation column (Milteny, Israel) and the CD8^+^ positive selection kit (Milteny, kit #130-045-201). Flow cytometry analysis using mouse anti-human CD8 (clone RTF-8, Southern Biotech) was performed to confirm that at least 95% of the purified cells were CD8 positive, mouse IgG1 (clone 15H6, Southern Biotech) was used as isotype control. Cells were pelleted and resuspended in 350 μl RNAprotect cell reagent (Qiagen, The Netherlands).

### RNA library preparation and sequencing

Cells were lysed using QIAshredder columns (Qiagen) and RNA was isolated using RNAeasy mini columns (Qiagen) according to the manufacturers protocol. The RNA concentration was measured on the Nanodrop ND-1000 Spectrophotometer (Thermo Fisher Scientific Inc., Norway). A random selection of RNA samples was checked for integrity using an Agilent 2100 Bioanalyser (Agilent, UK) yielding RIN-values above 7.0. 100 ng of RNA was processed using the TruSeq stranded mRNA library preparation kit # RS-122-2001 (Illumina, United States of America) according to the manufacturers protocol. Indexed libraries were sequenced by multiplexing 12 barcoded libraries per lane on an Illumina NextSeq using a 75 bp paired-end sequencing run. In total four sequencing runs were performed, with 4 samples undergoing library preparation and sequencing in duplicate.

### Data preprocessing

Using “kallisto”^20^ the fastQ-files were aligned to the “HomoSapiens.GRCh38.cDNA” transcriptome which was downloaded from the Ensembl FTP-server. Quality controls of the resulting mapped reads and differential gene expression analysis were performed in R3.4.2. Per-sample-per-transcript read counts were loaded into the DESeq2 package^21^ using the “tximport” function of the “tximport” package.^22^ To identify putative outlier samples, a Euclidean distance matric was calculated for all transcripts with at least 50 observations in every sample. Surrogate variable analysis was performed using the “svaseq” package^23^ in R to account for hidden confounders in the data. Only genes with at least one read in more than half the analyzed samples were kept for differential expression analysis. Furthermore, genes in the HLA region were excluded to avoid potential mapping issues given the hypermorphic nature of these genes.^24^

### Differential expression analysis

The “DeSeq2” package in R was used for differential expression analysis. The design matrix included surrogate variables identified through the SVA-package in addition to the case-control status. In order to account for multiple testing, we applied a false discovery rate correction using the option “Benjamini and Hochberg” in the DeSeq2 package. Adjusted p-values below 0.05 were considered significant.

### Testing for enrichment of nominally significant genes in GWAS, CD4^+^ T cell gene expression and CD8^+^ T cell protein expression studies

From the annotation provided for the 200 non-HLA genetic variants associated to MS,^1^ 272 gene IDs were extracted. From the genes expressed in the current CD8^+^ T cell data set, the MS-associated proportion of those that were not significantly (nominal p-value >0.05) differentially expressed was compared against those that were significantly (nominal p-value ≤0.05) differentially expressed using Fishers’ exact test.

We compared the genes that were nominally differentially expressed in our earlier gene expression study of CD4+ T cells^18^ against those that were nominally significantly differentially expressed in the current CD8^+^ T cell gene expression data. We only considered genes that were expressed in both cell types. Fishers’ exact test was used to test for significant enrichment or depletion of genes that were significant or not significant in both datasets.

Similarly, we compared genes encoding proteins that were nominally significant differentially expressed in an independent proteomics analysis that included CD8^+^ T cells from 13 MS patients and 14 healthy controls^25^ against the genes nominally significant in the current analysis. Fishers’ exact test was used to test for significant enrichment or depletion of genes that were significant or not significant in both datasets.

### Pathway analysis

Genes with adjusted p-values below 0.05; 0.1 and 0.4 in the differential expression analysis were imported into QIAGEN’s Ingenuity® pathway Analysis software (IPA®, QIAGEN, Redwood City, CA, USA, version 45868156, build version: 4,84,108 M). The input for these three pathway analyses were the differential expression ratios and associated p-values for the respective genes. Default analysis settings were used with the following confidence for species and tissues and cells, “*Mouse OR Rat OR Human*” (species) and “*only T cells (primary and cell-lines*)” (tissues/cell lines). Correction for multiple testing was done accordingly using the setting “*Benjamini & Hochberg*”.

Targeted pathway analysis for pathway dysregulation scores^26^ were performed for 18 pathways involved in T cell homeostasis (Supplementary Table S1), these pathways were selected from the gene set enrichment analysis database.^27^ Pathways that contained the term “GO_T_CELL*” were analysed for differences between MS patients and controls using the R package “pathifier”.^26^

## Results

A summary of the characteristics of the patients and healthy controls in this study is provided in Table 1. Detailed characteristics are provided in Supplementary Table S2. The mean number of mapped reads was 32.3 million (range 13.1–62.8 million). Duplicate samples showed highly similar profiles based on the Euclidean sample distance (Supplementary Figure S1). Duplicated samples were merged into one pseudo-sample in further analyses using the “collapseReplicates” function of the DeSeq2 R package.

**Table 1.** Basic characteristics of MS patients and healthy controls.

	N	Women (%)	Mean Age (SD)	Median EDSS (range)	Mean MS duration in years (range)
Patients	20	20 (100%)	35.6 (7.8)	2 (0.5–5)	7.3 (0.5–29)
Controls	20	20 (100%)	39.5 (7.3)	N/A	N/A

EDSS: expanded disability status scale.

**Figure 1.**
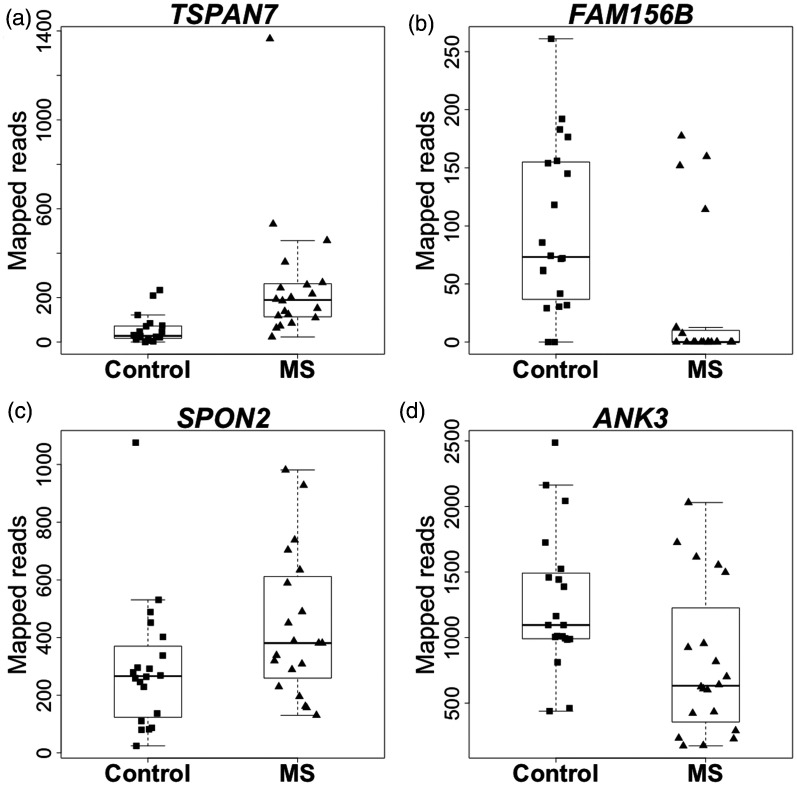
Boxplots of significantly differentially expressed genes with absolute fold-changes above 2 in CD8^+^ T cells from MS patients compared to healthy controls, ordered by the most significant genes. Individual expression levels are presented for healthy controls (squares) and MS patients (triangles). The boxes delimit 25% and 75% of the values; the horizontal bars represent the median value. The whiskers represent values that do not exceed a distance of 1.5 times the interquartile range from the middle 50% of the data. (a) The *TSPAN7* (Tetraspanin 7) gene which has higher expression in MS patients as compared to controls. Expression of this gene is close to zero or zero for the majority of the control samples (b) The *FAM156B* (Family With Sequence Similarity 156 Member B) gene which has higher expression in controls as compared to MS patients. Expression of this gene is close to zero or zero for the majority of the MS samples. (c) The *SPON2* (Spondin 2) gene which has higher expression in MS patients as compared to controls. (d) The *ANK3* (Ankyrin 3) gene which has higher expression in controls as compared to MS patients.

We performed differential gene expression analysis with multiple testing correction according to Benjamini and Hochberg (results of this analysis are provided in Supplementary Table S3). 36 genes reached an adjusted p-value below 0.05 (Table 2). Four genes displayed absolute fold-changes above 2 (*bold* print in Table 2), of which two genes had higher expression and two genes had lower expression in the MS patients’ CD8^+^ T cells (Figure 1). Closer inspection of the number of reads per gene revealed that some of the significantly differentially expressed genes are expressed at low levels, or not at all in most samples (exemplified by *TSPAN7* and *FAM156B* in Figure 1(a) and (b) respectively). Thirty of the 36 significantly differentially expressed genes were expressed at higher levels in MS patients, significantly more than can be expected by chance (Supplementary Table S4A, chi-square p-value 1.7*10^−5^). The excess of higher expressed genes is also observed for the more liberal p-value cutoffs of 0.1 and 0.4 for the differential expression analysis (Supplementary Tables S4B and S4C), whereas no significant excess of higher expressed genes is observed when considering a p-value cutoff of 0.9 (Supplementary Table S4D).

**Table 2.** Genes reaching a significant Benjamini-Hochberg corrected p-value in the differential gene expression analysis of CD8^+^ T cells from MS patients and healthy controls.

Gene	p-value	Adjusted p-value^a^	fold-change^b^	Absolutefold-change^c^	MS-associated SNP at locus^d^
*NOG*	1.04E-07	0.001	1.70	1.70	
*PIK3IP1*	2.36E-07	0.001	1.31	1.31	
*CHST12*	1.18E-06	0.004	1.41	1.41	rs55858457
***TSPAN7***	**1.61E-06**	**0.004**	**4.42**	**4.42**	
*GTPBP6*	1.32E-06	0.004	1.24	1.24	
*CYBC1*	2.12E-06	0.004	1.35	1.35	
*CTBP1*	2.66E-06	0.004	1.25	1.25	
*ABHD17A*	5.20E-06	0.008	1.34	1.34	
*S1PR5*	9.26E-06	0.012	1.67	1.67	
*TBXA2R*	1.22E-05	0.013	1.56	1.56	
*MGAT1*	1.23E-05	0.013	1.21	1.21	
*SP140*	1.45E-05	0.014	0.81	1.24	rs35540610
*EME2*	2.01E-05	0.018	1.61	1.61	
*ABI3*	2.27E-05	0.018	1.30	1.30	
***FAM156B***	**2.38E-05**	**0.018**	**0.03**	**35.34**	
*PRKAR1B*	3.06E-05	0.021	1.31	1.31	
*H2AX*	3.16E-05	0.021	1.65	1.65	
***SPON2***	**3.60E-05**	**0.023**	**2.07**	**2.07**	
*ZNF516*	3.98E-05	0.024	1.54	1.54	
*C6orf120*	4.69E-05	0.027	1.39	1.39	
*S1PR4*	5.06E-05	0.028	1.31	1.31	
*BCL11B*	6.42E-05	0.034	1.31	1.31	
*RNF126*	6.79E-05	0.034	1.28	1.28	
*RUNX3*	7.58E-05	0.036	1.18	1.18	rs6672420
*JUND*	8.10E-05	0.036	1.42	1.42	
*MIDN*	8.52E-05	0.036	1.36	1.36	
*H3C13*	8.21E-05	0.036	1.55	1.55	
*RBM38*	8.90E-05	0.037	1.29	1.29	
*SBF1*	1.01E-04	0.039	1.18	1.18	
*CTSD*	1.05E-04	0.039	1.21	1.21	
*THUMPD3*	1.02E-04	0.039	0.88	1.13	
*DDT*	1.22E-04	0.043	1.32	1.32	
***ANK3***	**1.27E-04**	**0.043**	**0.42**	**2.39**	
*PUF60*	1.31E-04	0.043	1.26	1.26	
*SLX1B*	1.31E-04	0.043	0.66	1.52	
*MMUT*	1.43E-04	0.046	0.81	1.24	

^a^Benjamini and Hochberg corrected p-value.

^b^Fold-change of gene expression for MS patients compared to healthy controls.

^c^Fold-change of gene expression between MS patients and controls, irrespective of the direction of the change.

^d^rs-ID of SNP that was identified at this gene locus in the latest IMSGC association study.^1^

### Gene pathway analyses

In order to investigate whether differentially expressed genes were enriched in specific pathways, Ingenuity Pathway Analysis was used for analyses for the 36 genes that were significant, as well as for genes that reached an adjusted p-value below 0.1 (83 genes) and 0.4 (530 genes). These genes and their adjusted p-values are provided in Supplementary Table S3. We did not observe significant enrichment in specific biological pathways (Supplementary Table S5-S7). After correction for multiple testing, pathways in the GSEA database that contain the terms “GO_T_CELL_*”, including “GO_T_CELL_ACTIVATION” did not show significant pathway dysregulation scores when comparing the MS patient scores against the controls (Supplementary Table S8).

### Analysis of MS susceptibility genes

The IMSGC recently published a genomic map for MS, which identified 200 non-HLA single nucleotide polymorphisms (SNPs) with association to MS.^1^ We investigated whether genes most proximal to these MS-associated SNPs were overrepresented among the differentially expressed genes in the CD8^+^ T cell dataset. Both for the comparison of the nominally significant genes (25 genes with MS-associated SNP, Supplementary Table S3) as well as for the multiple testing corrected significant genes (3 genes with MS-associated SNP, Table 2), we observed a higher number of MS-susceptibility genes than expected by chance (comparison provided in Table 3).

**Table 3.** Two-by-two table of genes reaching a multiple testing corrected p-value below 0.05 (or nominal p-values below 0.05 between brackets) in the differential gene expression analysis of CD8^+^ T cells from MS patients and healthy controls, divided into genes that are located in MS risk loci according to the latest GWAS study by the IMSGC.^1^ Statistical testing of significance was performed according to Fishers’ exact test.

	Genes not in an MS-risk locus^a^	Genes in an MS-risk locus^a^
Not differentially expressed^b^	17,119 (15,752)	202 (180)
Differentially expressed^b^	33 (1,400)	3 (25)
**Fisher exact test p-value**	**0.01 (0.04)**	

^a^Genes annotated to the IMSGC GWAS significant SNPs.^1^

^b^Genes with Benjamini-Hochberg corrected p-values ≤0.05 in the differential expression analysis (nominal p-values ≤0.05).

### Analysis of CD4^+^ T cell differential gene expression analysis data

We previously analyzed RNA sequencing data of CD4^+^ T cells of MS patients and controls.^18^ In order to identify possible overlap between differentially expressed genes we compared these datasets. For this, we only considered genes that were expressed by both cell types (indicated in Supplementary Table S3). We analyzed whether there was more overlap than expected by chance for genes reaching a nominally significant differential expression. Amongst the 11,902 genes expressed in both CD4^+^ and CD8^+^ T cell we observed no significant overlap of genes that reach nominal significance in either dataset (Fishers’ exact test p-value 0.26, Supplementary Table S9A).

### Analysis of CD8^+^ T cell protein differential expression analysis data

We previously analyzed the protein expression profile of CD8^+^ T cells from MS patients and healthy controls.^25^ We compared the genes encoding the proteins in this differential expression analysis against the differential expression of genes in the current study. We only considered genes detected in both datasets (2,113 genes). No significant enrichment of nominally differentially expressed genes/proteins was observed in this comparison (Fishers’ exact test p-value 0.07, Supplementary Table S9B).

## Discussion & conclusion

We show that gene expression of CD8^+^ T cells is significantly different between MS patients and healthy controls. We identified 36 genes that are significantly differentially expressed upon multiple testing correction. Four of these genes have a more than 2-fold difference in expression between MS patients and healthy controls. A striking majority of the differentially expressed genes have higher levels of expression in MS patients compared to the healthy controls. The predominant higher expression of differentially expressed genes may be attributable to a more active state of the CD8^+^ T cells. However, we did not identify specific T cell activation pathway signatures amongst the differentially expressed genes that can confirm this hypothesis. Further investigation of the activation status and potential of CD8^+^ T cells obtained from MS patients and healthy controls should be performed in order to conclude whether or not activation state underlies the observed higher expression of the majority of differentially expressed genes in CD8^+^ T cells from MS patients.

Amongst the differentially expressed genes that are nominally significant and those that remain significant after multiple testing correction, there was a significant enrichment of genes that were implicated in the latest large-scale GWAS.^1^ It should be noted that the annotation of GWAS results to the underlying genes is an ongoing process and it is likely that not all the annotated genes at these loci are relevant for MS. This would however only lower our power to detect a significant enrichment. We therefore conclude that genes that are differentially expressed in CD8^+^ T cells of MS patients are more likely to be at MS-associated genetic loci.

Although patients were not treated for MS at the time of inclusion in this study, we sampled these patients after disease onset. Therefore, we cannot exclude that the observed differences are an indirect consequence of the disease activity. Whether the gene expression of CD8^+^ T cells plays a role in the onset of the disease remains to be investigated in a different study design. Furthermore, a larger sample size of healthy controls and patients with different disease durations, or a collection of patients that have samples available at multiple timepoints is needed in order to better evaluate the impact of disease duration on the gene expression differences. Similarly, this study is not sufficiently powered to assess whether specific clinical parameters explain outlier samples observed in some of the significantly differentially expressed genes (e.g. outlier samples are observed in *TSPAN7* and *SPON2* in Figure 1(a) and (c) respectively). Given the relatively small differences in gene expression observed in this study, validation should be performed in an independent study population.

Pathway analyses of differentially expressed genes with different p-value cutoffs, did not point out specific biological pathways that are affected in the MS patients. A targeted pathway analysis for T cell pathways did not reveal differences in the dysregulation scores^26^ for these between the patients and controls. It should be noted that biological pathways that are not currently annotated in the databases used are not included in the pathway analyses performed, therefore the possibility remains that there are biological pathways in CD8^+^ T cells that are affected by MS.

Comparison of this analysis against an earlier differential gene expression study of CD4^+^ T cells from 20 MS patients (17 included in the current study) and 19 healthy controls (all included in the current study) did not show a significant overlap of results, indicating that the observed changes in gene expression may not be observed in other circulating T cells. Furthermore, comparison of the differential gene expression analysis of CD8^+^ T cells against an earlier differential protein expression analysis of CD8^+^ T cells did not show overlap in nominally significant genes in these studies. It should be noted that both the current gene expression study and the earlier protein expression study have relatively low power. Furthermore, due to additional regulatory steps between the transcription into RNA and translation to protein, the correlation between gene expression and protein expression is not linear and influenced by many additional factors for a large number of genes.^28^ It remains to be further investigated how differences in gene expression in CD8^+^ T cells from MS patients relate to putative differences in protein expression.

The most significant differentially expressed gene*, NOG*, encodes the protein *noggin*. Although there is no role shown in immune cells, this protein is hypothesized to be involved in remyelination of MS lesions.^29^ It remains to be investigated what biological mechanism results in the upregulation of this gene in CD8^+^ T cells. Two of the four genes with at least a 2-fold change in MS patients have almost no expression in either patients (*FAM156B*) or controls (*TSPAN7*). Whether this is a reflection of respectively down- or upregulation, or the result of different CD8^+^ T cell subsets exhibiting different gene expression signatures remains to be investigated in more detailed gene expression analyses of CD8^+^ T cells of MS patients and subsets of CD8^+^ T cells. TSPAN7 is a predicted as a top protein-protein interaction partner for TSPAN32; the *TSPAN32* gene was shown to be less expressed on MS patients’ CD4^+^ T cells upon *in vitro* activation.^30^ *SPON2* expression was previously shown to be higher expressed in CD8^+^ T cells during acute infectious mononucleosis (AIM) as compared to the same patients in convalescence.^31^ AIM is usually caused by the Epstein-Barr virus, which is a known risk factor for developing MS.^32^ *ANK3* is an established risk gene for psychiatric illness,^33^ whilst it has not previously been linked to autoimmune diseases. For *FAM156B* there is little known about the role of the encoded protein. Amongst the remaining genes that were significantly differentially expressed, we observed two genes belonging to the Sphingosine-1-Phosphate Receptors (*S1PR4* and *S1PR5*), which are targeted by the highly effective MS drug Fingolimod. The functional mechanism of Fingolimod is the sequestering the lymphocytes in lymph nodes, thereby preventing these from entering the CNS which in turn may prevent relapses. The *S1PR4* and *S1PR5* genes are expressed by lymphocytes and regulate lymphocyte egression from lymph nodes.^34^ For both genes we observed a significantly higher expression for the MS patients.

This study was performed on purified CD8^+^ T cells, which provides a detailed insight in the gene expression of these specific cells. It should however be noted that CD8^+^ T cells can be divided further into subclasses. This leaves the possibility open that some or all of the observed differential gene expression is attributable to different proportions of CD8^+^ T cell subsets. This could be overcome by future experiments that include flow cytometry analysis of proteins of interest, combined with cell type markers or experiments that apply single cell RNA sequencing, in which each individual cell can be traced back from the sequencing data and subdivisions can be made based on signature genes of subsets of larger cell populations. The patients in this study were untreated at the time of inclusion, which overcomes potential immune system changes induced by MS medications. Whilst some patients were recently diagnosed, others had been diagnosed years before the inclusion. In general, these patients were benign based on their expanded disability status scale (EDSS) scores at the time of inclusion. These patients were included in this study during remission; it remains possible that sampling during a relapse will provide additional insights in differential gene expression of MS patients’ immune cells compared to healthy controls.

In conclusion, our study shows that CD8^+^ T cell gene expression is different in MS patients as compared to controls. Whether this is the result of different active processes in the T cells of MS patients, or the result of different subsets of CD8^+^ T cells for MS patients remains to be investigated.

## Supplemental Material

sj-pdf-1-mso-10.1177_2055217320978511 - Supplemental material for CD8^+^ T cell gene expression analysis identifies differentially expressed genes between multiple sclerosis patients and healthy controls Click here for additional data file. Supplemental material, sj-pdf-1-mso-10.1177_2055217320978511 for CD8^+^ T cell gene expression analysis identifies differentially expressed genes between multiple sclerosis patients and healthy controls by IS Brorson, AM Eriksson, IS Leikfoss, V Vitelli, EG Celius, T Lüders, T Berge, HF Harbo, H Nilsen and SD Bos in Multiple Sclerosis Journal—Experimental, Translational and Clinical

sj-xlsx-2-mso-10.1177_2055217320978511 - Supplemental material for CD8^+^ T cell gene expression analysis identifies differentially expressed genes between multiple sclerosis patients and healthy controls Click here for additional data file. Supplemental material, sj-xlsx-2-mso-10.1177_2055217320978511 for CD8^+^ T cell gene expression analysis identifies differentially expressed genes between multiple sclerosis patients and healthy controls by IS Brorson, AM Eriksson, IS Leikfoss, V Vitelli, EG Celius, T Lüders, T Berge, HF Harbo, H Nilsen and SD Bos in Multiple Sclerosis Journal—Experimental, Translational and Clinical

## Data access

For the purpose of MS-related research, requests to access raw data in this study can be addressed to grants@ous-hf.no.
