# Supplementary material for: CD8+ T cell gene expression analysis identifies differentially expressed genes between multiple sclerosis patients and healthy controls
Source: Mult Scler J Exp Transl Clin. 2020 Dec 9;6(4):2055217320978511. doi: 10.1177/2055217320978511 (PMC7731718; doi:10.1177/2055217320978511)
Supplement: sj-pdf-1-mso-10.1177_2055217320978511 - Supplemental material for CD8+ T cell gene expression analysis identifies differentially expressed genes between multiple sclerosis patients and healthy controls [file sj-pdf-1-mso-10.1177_2055217320978511.pdf]

Supplementary Figure S1

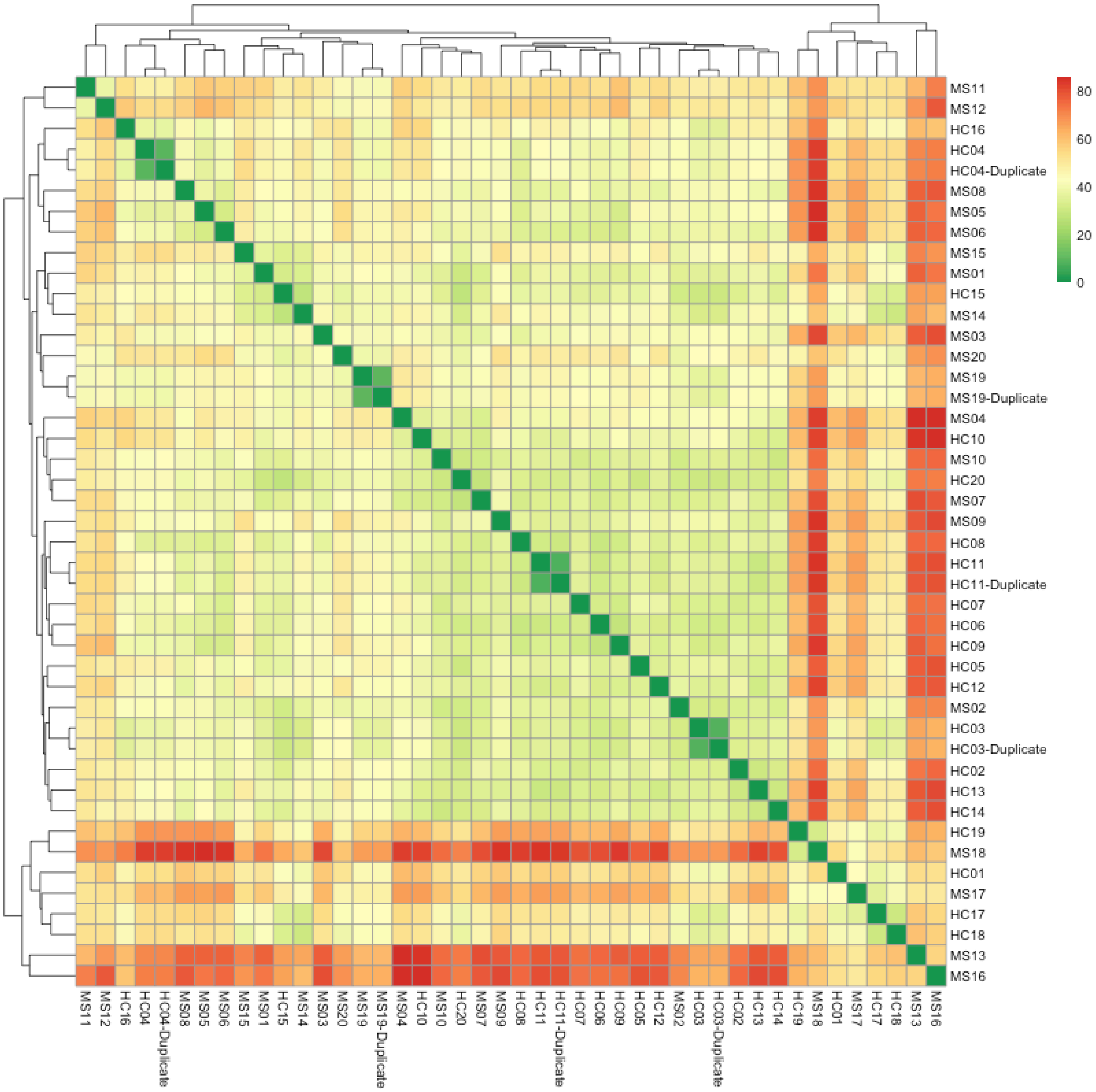

Supplementary Figure S1. Heatmap of the Euclidean distance between all samples. The distance matrix is based on all genes with at least one observed read in half of the samples. Sample duplicated are indicated in the right-hand axis. All duplicate samples show high levels of similarity (green boxes). Eight samples cluster slightly away from the remaining 36 samples, this cluster is not defined by the case control status (it contains both MS patients and healthy controls).
